# Effect of fermented oyster (*Crassostrea gigas*) extracts and regular walking on muscle strength and mass in older adults with relatively low muscle mass: A randomized controlled trial

**DOI:** 10.3389/fnut.2022.935395

**Published:** 2022-07-25

**Authors:** Ye Li Lee, Sang Yeoup Lee

**Affiliations:** ^1^Integrated Research Institute for Natural Ingredients and Functional Foods, Yangsan, South Korea; ^2^Family Medicine Clinic and Biomedical Research Institute, Pusan National University Yangsan Hospital, Yangsan, South Korea; ^3^Department of Medical Education, Pusan National University School of Medicine, Yangsan, South Korea

**Keywords:** fermented oyster, *Crassostrea gigas*, muscle, strength, dietary supplements, sarcopenia, randomized controlled trial

## Abstract

**Introduction:**

Oysters possess an excellent nutritional profile containing γ-aminobutyric acid (GABA). Previous data suggest that GABA is a potent bioactive compound for improving muscle health. Lactic acid fermentation is thought to increase GABA content. However, the effect of oral supplementation of fermented oyster extracts (FO) on human muscle strength and mass is unclear. Therefore, we tested the effects and safety of consumption of FO combined with regular walking for 12 weeks on muscle strength and mass in older adults with relatively low muscle mass.

**Materials and methods:**

A randomized controlled trial was conducted on 54 adults between 50 and 78 years of age. Participants were randomized to receive either placebo or 1,200 mg FO daily for 12 weeks. By fermentation with *Lactobacillus brevis* BJ20, FO was prepared from *Crassostrea gigas*. At baseline and at 12 weeks after treatment, the following parameters of the participants were examined: knee strengths, handgrip strengths, body composition, blood tests, and 24-h dietary recall. All participants were required to walk for 30–60 min/day for >3 days/week during the trial period. Physical activity was assessed using an exercise log during the study.

**Results:**

Of the 54 participants, 49 completed the trial without reporting adverse effects. FO supplementation over 12 weeks did not cause any increase in knee or grip strength compared to the control group. Also, no differences were observed in the muscle mass, growth hormone, muscle biomarkers, anti-inflammatory markers, and antioxidative markers between the two groups. None of the participants experienced adverse events. Application of FO was well tolerated, and no notable adverse effect was reported in both groups.

**Discussion:**

FO supplementation with regular walking did not improve remarkably muscle function compared to regular walking alone in adults with relatively low muscle mass.

**Clinical Trial Registration:**

[www.ClinicalTrials.gov], identifier [NCT04109911].

## Introduction

Muscle mass and muscle strength reach a peak in the mid-20s; then onward, muscle mass gradually decreases with reduced physical activity and altered protein metabolism, and muscle strength decline is almost 3–5 times greater than the loss of muscle mass. This phenomenon is greater in men than in women. Furthermore, this is associated with a decrease in physical function and is itself strongly associated with morbidity and mortality (1). Skeletal muscle is a dynamic tissue with excellent regenerative ability and remarkable plasticity to adapt to various external stimuli such as external stimulation, intrinsic factors, or physical activity (2). Insulin-like growth factor-1 (IGF-1) plays a key role as a growth factor that regulates both muscle protein synthesis and degradation *via* multiple mechanisms such as ubiquitin-proteasome system, phosphatidylinositol 3 kinase/Protein kinase B/mammalian target of rapamycin and phosphatidylinositol 3 kinase/Protein kinase B/glycogen synthase kinase β pathways, and autophagy. IGF-1 also activates muscle stem (satellite) cell proliferation in muscle regeneration (2, 3). It is known that growth hormone release and IGF-1 expression are stimulated directly or indirectly by γ-aminobutyric acid (GABA) (4, 5).

Oysters contain a large amount (4.8%/dry base) of glutamic acid, a precursor of GABA. They also contain a large amount of phosphorus taurine (based on a 4.2%/dry base) and represent one of the world’s top 10 seafood foods rich in essential minerals such as glycogen, vitamins, iron, and iodine (6). Recently, the effects of lactic acid-fermented oyster extract (FO) containing GABA and lactic acid, produced by lactic acid fermentation of oysters, on bone formation (7), height growth (8), and exercise performance improvement (9) have been reported. Also, a previous study showed that GABA-enriched FO reduced muscle proteolysis by increasing mRNA levels of IGF-1 expression in dexamethasone-treated animals (10). These data suggest that GABA is a potent bioactive compound for improving muscle health (11). Lactic acid fermentation may further increase GABA content (9, 10).

FO supplementation was therefore expected to improve sarcopenia. In a pilot study of postmenopausal women, 1.0 g FO supplementation every 8 weeks resulted in greater increase in the knee flexor strength than placebo. In addition, the concentrations of IGF-1 and human growth hormone increased in the FO group compared with those in the control group (11). Based on previous studies, we hypothesized that FO has a beneficial effect on preserving and strengthening muscle strength and function in older adults. Therefore, this randomized, double-blind, placebo-controlled trial aimed to investigate the impact of 12 weeks of daily FO (*Crassostrea gigas*) supple-mentation and regular walking on muscle mass and strength in older adults (both men and women) who have a low muscle mass.

## Materials and methods

### Study design and ethical aspects

The study was designed as a randomized, placebo-controlled, double-blind clinical trial. All subjects gave their informed consent for inclusion before they participated in the study. The study was conducted in accordance with the Declaration of Helsinki, and the protocol was approved by the Ethics Committee of the Institutional Review Board at Pusan National University Yangsan Hospital (IRB No. 02-2019-013, July 1, 2019). This trial is registered with ClinicalTrials.gov (NCT04109911, 01/10/2019).

### Study participants

Study candidates were recruited through an advertisement at a tertiary hospital in Yangsan-si, South Korea. Participants aged between 50 and 85 years, with body mass index (BMI) ranging from 18.5 to 30.0 kg/m^2^ and with relatively low skeletal muscle mass (<110% of the standard lean mass measured using the body composition analyzer InBody 720) were eligible for the study. Participants with an abnormal liver or renal function (aspartate aminotransferase or alanine aminotransferase level ≥60 IU/L, creatinine level ≥1.2 mg/dL, or proteinuria, defined as urinalysis dipstick reading of ≥2 +), with uncontrolled diabetes (fasting glucose level ≥160 mg/dL), with uncontrolled hypertension [blood pressure (BP) ≥160/100 mmHg], or notable cardiac diseases such as angina or myocardial infarction, history of a gastrectomy, known allergies, and those on medications for psychiatric illness or any herbal treatments within the past 2 months were excluded. The subjects were considered to have dropped out or discontinued participation in the trial and were excluded from the per-protocol analysis according to the following criteria: failure to take the test product or placebo for 5 consecutive days, failure to attend follow-up assessments, and poor compliance (<80%). The participants were requested to log supplement intake in a diary whenever they took the supplement in a diary, which was turned in along with the bottle to the researcher at every visit. Compliance was assessed by counting the remaining capsules at every visit. Twelve participants met the exclusion criteria.

### Randomization

**FIGURE 1:**
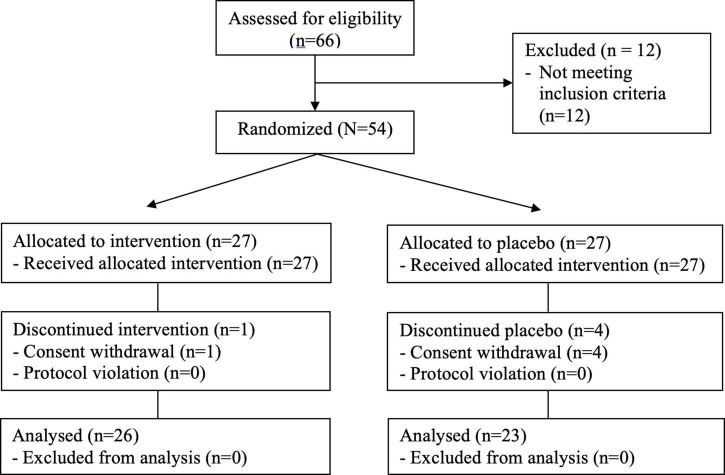
CONSORT flow diagram.

Sixty-six participants were recruited for screening and 54 (81.8%) participants were finally enrolled after undergoing baseline measurements. They were randomly assigned to either one of the intervention group with FO (IG) or the control group with placebo (CG) through block randomization using randomized numbers and given identification numbers on recruitment. The IG (*n* = 27) received 1,200 mg of FO supplement (400 mg each capsule) per day. The CG (*n* = 27) received a placebo (Figure 1). Randomization codes were created by an expert in statistics using nQuery Advisor 7.0. Those who were responsible for deciding on study eligibility and conducting the measurements were kept unaware of the results of the randomization throughout the whole study process. Every participant was asked to visit the center four times in total (visit 1, for screening; visit 2, for randomization and starting supplementation; visit 3, 6 weeks after the intervention; visit 4, 12 weeks later).

### Test product and placebo

FO extract was obtained from Marine Bioprocess Co., Ltd. (Busan, Republic of Korea). The participants were randomly assigned to the FO group or the placebo group. The FO group was administered 1,200 mg FO/day orally, i.e., one 400 mg capsule 30 min after breakfast, lunch, and after dinner, for 12 weeks. In our previous study (11), after FO was dissolved in distilled water and analyzed according to the official analytical methods used for the specifications stated in the food code (12), the content of GABA, a bioactive compound, was 1,053.7 mg per 100 g of FO (11). The content of other amino acids was as follows: arginine (6,183.3 mg/100 g) and phenylalanine (217.9 mg/100 g), which are essential amino acids, and leucine (122.6 mg/100 g), isoleucine (59.8 mg/100 g), and valine (16.4 mg/100 g), which are also known as branched-chain amino acids (BCAA). Citruline, a precursor for the synthesis of arginine, was present at a concentration of 221.4 mg/100 g (11). The proportions of each component in 1,200 mg of FO fermented with *Lactobacillus brevis*. BJ20 as follows: GABA (11.2%), lactic acid (5.0%), water (5.5%), protein (31.8%), and carbohydrate (56.1%) (11). The placebo group was administered the same quantity of placebo as the FO. The placebo was identical to the FO capsule in appearance and was filled with corn starch. Based on the results of a previous animal study, which showed that the minimum efficacious and safe dose of FO was 200 mg/kg (10), the dose used in the animal subjects was converted to a human equivalent dose based on the person’s body surface area, i.e., 960 mg for individuals weighing 60 kg. In addition, a previous preliminary clinical study had established the safety of FO extracts at 1,000 mg/day (11). Thus, we selected 1,200 mg/60 kg as the final dose considering manufacturing and intake convenience.

### Measurements of efficacy

The primary outcome measure was the change from the baseline in the peak torque (TQ) at 60°/s of knee extension/flexion representing muscle function at 12 weeks of FO or placebo use. The secondary outcome measures were changes in the handgrip strength, body composition, creatinine, pyruvate, and lactate (13), and the Euro-QoL-5D (EQ-5D) score, which is representative of the quality of life (14), during the 12-week treatment period.

#### Biomarkers measurements

All laboratory analyses were conducted in the hospital clinical laboratory. Blood samples, after a 12-h overnight fast, were collected at baseline and at 12 weeks after the randomization to evaluate the biomarkers of muscle metabolism and monitor the potential adverse effects of FO. The serum creatinine was measured using modified Jaffe’s kinetic alkaline picrate method. Pyruvate was measured by the enzymatic method on AU5800 chemistry analyzer (Beckman Coulter, Brea, CA, United States) with an inter-and intra-assay coefficient of variation (CV) of 2.7 and 3.0%, respectively. Lactate concentrations were measured by ion-selective electrode assay using Stat Profile pHOx Ultra analyzer (Nova Biomedical, Waltham, MA, United States), which had an inter-and intra-assay CV of 3.0 and 5.0%, respectively.

#### Body composition analysis

Body composition analysis was determined using the whole-body dual-energy X-ray absorptiometry (Hologic Horizon W, Software: Apex Versions 5.6.0.5, Hologic Inc., Marlborough, MA, United States) in the supine position as per the guidelines of the manufacturer. Appendicular lean mass (ALM) was defined as the total lean soft-tissue mass of four limbs. In this study, appendicular lean mass index (ALMI) and skeletal muscle mass index (SMI) were, respectively, estimated based on the following formulae: ALM in kg divided by height in meters squared and ALM in kg divided by weight multiplied by 100, respectively (15, 16).

#### Dietary intake and physical activities assessments

The participants were required to walk for 30 min^–1^ h each day, >3 times/week, and were counseled to maintain their usual diets during the 12-week period. The walking activity was assessed using a walking log during the study. At baseline and after12 weeks of the trial, the participants were asked to answer a questionnaire on physical activities and dietary intake that may affect the skeletal muscle status. Physical activity was assessed using the international physical activity questionnaires (17) on frequency, intensity, and type of physical activities during the previous 7 days and the number of physical activities was represented as the metabolic equivalent of task. Food intake was assessed using the 24-h dietary recall method. For nutrient analysis of the surveyed dietary intake, the CAN-Pro version 4.0 (Computer-Aided Nutritional Analysis Program for Professionals 4.0; Korean Nutrition Society) was used.

### Evaluation of safety and additional benefits

To evaluate the safety and additional benefits of FO, the participants’ BP and laboratory test results [complete blood cell count, concentration of liver enzymes, glucose, creatinine, insulin, free fatty acid, high-sensitivity C-reactive protein (hs-CRP) (a marker of micro-inflammation), creatine kinase (CK), and malondialdehyde (a marker of oxidative stress)] (18) were assessed. Blood pressure and heart rate were tested three times in the sitting position after a 10-min rest using a model BP-203 RV II device (Colin Corp., Aichi, Japan). The average measurement was recorded. Body weight and height were measured using a digital scale and stadiometer (BSM370; Biospace Co., Ltd., Seoul, South Korea), with patients wearing a light gown without shoes. Fasting glucose was measured using a glucose oxidase test method (LX-20, Beckman Coulter, Fullerton, CA, United States), hs-CRP was measured by latex particle-enhanced central immunoturbidimetric assay with an inter- and intra-assay CV of 5.0 and 10.0%, respectively, creatinine kinase was analyzed by the CK NAC-activated procedure, and liver enzyme concentrations were measured according to an enzymatic colorimetric method on AU5800 chemistry analyzer (Beckman Coulter, Brea, CA, United States). Free fatty acid was determined by an enzymatic colorimetric method assay (NEFA-HR2, ACS-ACOD; Wako Chemicals, Neuss, Germany) on Hitachi LABOSPECT 008 (Hitachi High-Tech Co., Tokyo, Japan), which had an inter-and intra-assay CV of 2.0 and 1.3%, respectively. Malondialdehyde concentration was assessed using the OxiSelect™ thiobarbituric acid reactive substances assay kit (Cell Biolabs Inc., San Diego, CA), which had an inter- and intra-assay CV of 2.0 and 2.0%, respectively. The relative fluorescence intensity of cells was estimated with the Varioskan Flash spectral scanning multimode reader (Thermo Fisher Scientific Inc., Waltham, MA). Serum insulin was quantified using chemiluminescent microparticle immunoassay on the Alinity i system (Abbott Laboratories, Abbott Park, IL, United States) with an inter- and intra-assay CV of 1.8 and 1.9%, respectively. Insulin resistance was assessed by calculating the Homeostatic Model Assessment for Insulin Resistance (HOMA-IR) using the formula (fasting glucose x fasting insulin)/405. Reports of any other adverse events or unpredicted allergic reactions were collected throughout the study.

### Statistical analyses

We used MedCalc version 19.4.1 (MedCalc Software Ltd., Ostend, Belgium) to calculate the sample size, which was based on a similar earlier study (19). The estimated sample size was 18 subjects per group for 80% power to detect a difference of 4.36 Nm mg/dL in average knee extension peak TQ at an angular velocity of 60°/s, assuming an SD of 4.6 Nm in the primary outcome variable and an α error of 5%. Then, the sample size was adjusted to 27 participants per group to allow for 30% dropouts. Intention-to-treat (ITT) analysis was primary for comparisons of outcomes between the FO and placebo groups, with multiple imputation of missing data (*n* = 54). Because the percentage of missing values at the 12-week follow-up was 11.3% for all variables, 5 imputed data sets were created and the results of the analyses from the different imputed data sets were pooled according to Rubin’s rules using R software version 3.6.2 (R Foundation for Statistical Computing). The multivariate imputation by chained equations algorithm was used with the predictive mean matching method. A per-protocol (PP) analysis was also performed (*n* = 49) to assess the effectiveness of the supplementation. Shapiro–Wilk’s test was employed to test the normality assumption. Intergroup comparisons of baseline characteristics were performed using the 2-sample *t*-test for continuous variables (or Mann-Whitney’s *U*-test for non-parametric continuous variables) and the chi-square test for categorical variables (or Fisher’s exact test for non-parametric categorical variables). ANCOVA or rank ANCOVA was used for the main analysis, with adjustments for age, sex, baseline variables, and the changes in dietary total caloric intake and physical activity from baseline to 12 weeks, all of which were calculated as a delta, i.e., 12-week-baseline as covariates. Model assumptions were checked by histograms, normal probability plots, and residual scatter plots. Safety analyses included all randomized patients who were exposed to at least one dose of study intervention. A *p*-value < 0.05 was considered statistically significant. Data were analyzed using IBM SPSS Statistics 22.0 (IBM Inc., Armonk, NY, United States) and R software version 3.6.2.^1^

## Results

### Consolidated standards of reporting trials flow diagram and baseline characteristics of the subjects

The flow of participants through the controlled interventional trial is depicted in a CONSORT (consolidated standards of reporting trials) conform diagram (20) (Figure 1). Fifty-four participants aged 50–78 years were statistically analyzed. The mean age in the IG (*n* = 27) was 58.1 ± 6.7 years and in the CG (*n* = 27), 59.3 ± 5.1 years. One participant in the IG and four in the CG withdrew consent for personal reasons that were not considered associated with the trial. The characteristics of these four participants were similar to those of the others who completed the study. Compliance exceeded 88% in both the IG and CG. Hence, ITT population and PP population were 54 and 49, respectively. Supplement adherence was 95.9 ± 4.6% in the IG and 95.4 ± 6.2% in the CG among the PP population. Mean ALMI (kg/m^2^) and SMI (%) of participants were 5.6 ± 0.8 (3.9–7.4) and 23.8 ± 3.2 (16.5–31.2), respectively. One participant in the FO group and four participants in the placebo group withdrew from the study due to personal reasons. Overall, 49 subjects (90.7%) completed the trial. Comorbid disorders were observed in eight (29.6%) (three: hypertension, three: diabetes, two: dyslipidemia, five: others) in the FO group and 11 (40.7%) (six: hypertension, three: diabetes, five: dyslipidemia, four: others) in the placebo group. Randomization was successful, as the two groups generated were comparable for all variables, and no significant differences were observed in the baseline demographic and anthropometric characteristics between the groups (Table 1). In the FO group, energy intake increased after 12 weeks compared to baseline in the PP analysis (*P* = 0.029). However, there were no significant differences in calorie intake and physical activities during the 12 weeks of the trial between groups, reflecting no additional effect that might have influenced skeletal muscle status aside from the intervention (Table 2). During the whole study period, the double-blind requirement was well maintained. In both groups, the compliance with walking (more than 3 times a week) was over 95%.

**TABLE 1:** Baseline characteristics of the study group.

Variables	Intention to treat population			Per protocol population		
	FO (*n* = 27)	Placebo (*n* = 27)	*p^a^*	FO (*n* = 26)	Placebo (*n* = 23)	*p^a^*
Age, years	58.1 ± 6.7	59.3 ± 5.1	0.441	58.2 ± 6.8	59.4 ± 5.3	0.494
Males, %	3 (11.1)	8 (29.6)	0.091	3 (11.5)	7 (30.4)	0.101
Systolic BP, mmHg	124.4 ± 13.9	126.9 ± 14.0	0.503	124.4 ± 14.2	126.6 ± 15.1	0.591
Diastolic BP, mmHg	78.5 ± 10.1	82.4 ± 10.3	0.162	78.2 ± 10.2	82.3 ± 11.1	0.184
Current smoker,%	1 (3.7)	0 (0.0)	1.000	1 (3.8)	0 (0.0)	1.000
Alcohol drinker, %	6 (22.2)	5 (18.5)	0.735	5 (19.2)	4 (17.4)	0.670
Co-morbidities*^b^*	8 (29.6)	11 (40.7)	0.393	8 (30.8)	10 (43.5)	0.357
Hypertension	3 (11.1)	6 (22.2)		3 (11.5)	6 (26.1)	
Diabetes mellitus	2 (7.4)	3 (11.1)		2 (7.7)	3 (13.0)	
Dyslipidemia	2 (7.4)	5 (18.5)		2 (7.7)	5 (21.7)	
Others	5 (18.5)	4 (14.8)		5 (19.2)	4 (17.4)	
Body mass index, kg/m^2^	23.6 ± 3.2	23.7 ± 2.1	0.861	23.3 ± 3.0	23.6 ± 2.2	0.728
Energy intake, Kcal/day	1,527.2 ± 428.1	1,495.1 ± 507.4	0.802	1,531.1 ± 436.1	1,515.4 ± 516.2	0.909
IPAQ (METs)	1,215.0 [583.1−2,709.0]	1,825.0 [749.3−3,445.3]	0.236	1,256.0 [600.0−2,772.0]	1,485.0 [618.8−3,443.3]	0.631

Values are mean ± SD or n (%), except for IPAQ, which are median [IQR] unless otherwise indicated. FO, Fermented Oyster Extract; BP, blood pressure; IPAQ, international physical activity questionnaires; MET, metabolic equivalent task. ^a^P-value by two-sample t-test for parametric variables, Mann-Whitney’s U-test for non-parametric variables, and the chi-square test or Fisher’s exact test for categorical variables. ^b^Including multiple diseases.

**TABLE 2:** Energy intake and physical activity between the two groups for 12 weeks.

	FO group		Placebo group		Adjusted difference of FO vs. placebo over 12 weeks	*p^a^*
	Baseline	12 week	Baseline	12 week		
**Intention to treat (*n* = 54)**						
Energy intake, Kcal/d	1527.2 ± 428.1	1968.7 ± 508.4	1495.1 ± 507.4	1433.1 ± 626.4	301.01 (−119.97, 722.13)	0.197
IPAQ, METs	1,215.0 [583.1−2,709.0]	1,278.0 [657.0−3,291.0]	1,825.0 [749.3−3445.3]	1,863.7 [693.0−3.666.0]	−	0.731
**Per protocol (*n* = 49)**						
Energy intake, Kcal/d	1531.1 ± 436.1	1751.1 ± 403.5*^b^*	1515.4 ± 516.2	1558.8 ± 361.3	−240.28 (−537.65, 57.09)	0.111
IPAQ, METs	1,256.0 [600.0−2,772.0]	1,216.5 [657.0−3,291.0]	1,485.0 [618.8−3,443.3]	1,633.5 [693.0−3,465.0]	−	0.583

Values are median [IQR] or mean (95% CI) unless otherwise indicated. FO, Fermented Oyster Extract; IPAQ, international physical activity questionnaires; MET, metabolic equivalent task. ^a^ANCOVA or rank ANCOVA adjusted for age, sex, and each baseline value as covariates over the 12-week period. ^b^P < 0.05 by paired t-test within each group.

### Primary outcome

At the 12-week, flexor strength performance of the right knee was significantly increased than that at the baseline in only the FO group (*p* < 0.001), based on ITT analysis (Table 3). At 12 weeks, flexor strength performances of both knees (right and left) and extensor strength performance of the left knee were significantly increased than those at the baseline in the FO group (*P* = 0.007, *P* = 0.002, and *P* = 0.023, respectively), while only extensor strength performance of both knees (right and left) was significantly increased than that at the baseline in the placebo group (*P* = 0.014 and *P* = 0.008, respectively), based on PP analysis (Table 3). However, Table 3 shows no intergroup difference in the strength performance of both knees after 12 weeks of treatment in both the PP and the ITT analysis.

**TABLE 3:** Primary outcome measures of the two groups.

	FO group		Placebo group		Adjusted difference of FO vs. placebo	*p^a^*
	Baseline	12 week	Baseline	12 week		
**Intention to treat (*n* = 54)**						
60°/s knee extension peak TQ (R), Nm	70.8 ± 26.9	78.4 ± 26.2	72.8 ± 29.2	80.5 ± 35.4	−4.66 (−23.99, 13.97)	0.593
60°/s knee extension peak TQ (L), Nm	72.8 ± 23.7	79.6 ± 25.0	75.1 ± 27.1	83.1 ± 37.3	−8.45 (−26.31, 9.41)	0.400
60°/s knee flexion peak TQ (R), Nm	23.1 ± 11.0	31.0 ± 12.7*^b^*	26.8 ± 15.4	30.8 ± 16.0	3.08 (−7.14, 13.31)	0.630
60°/s knee flexion peak TQ (L), Nm	25.2 ± 9.2	29.4 ± 13.5	28.9 ± 13.3	28.3 ± 17.2	4.25 (−6.10, 14.59)	0.437
**Per protocol (*n* = 49)**						
60°/s knee extension peak TQ (R), Nm	70.9 ± 27.5	80.1 ± 21.5	73.6 ± 31.0	81.5 ± 30.1*^c^*	−4.11 (−16.95, 8.74)	0.671
60°/s knee extension peak TQ (L), Nm	72.3 ± 24.0	79.9 ± 22.6*^c^*	76.7 ± 28.4	84.4 ± 29.2*^c^*	−7.71 (−18.36, 2.94)	0.823
60°/s knee flexion peak TQ (R), Nm	22.9 ± 11.1	31.8 ± 11.7*^b^*	26.8 ± 16.7	30.1 ± 13.7	4.26 (−4.25, 12.77)	0.319
60°/s knee flexion peak TQ (L), Nm	24.6 ± 8.9	30.1 ± 11.8*^c^*	29.3 ± 14.0	27.9 ± 13.7	5.20 (−3.82, 14.22)	0.252

Values are mean ± SD or mean (95% CI) unless otherwise indicated. FO, Fermented Oyster Extract; TQ, torque. ^a^ANCOVA adjusted for age, sex, and each baseline value over the 12-wk period. ^b^P < 0.005, ^c^P < 0.05 by paired t-test or Wilcoxon signed-rank test within each group.

### Secondary outcome

Among the secondary outcome variables in the PP analysis, only ALMI was increased at 12 weeks than that at the baseline in only the FO group (*P* = 0.038, Table 4). However, all secondary outcomes including handgrip strength, ALMI, AMI, body fat percent, creatinine, pyruvate, lactate, and quality of life scores did not differ between the two groups throughout the study period, based on both the PP and the ITT analysis (Table 4).

**TABLE 4:** Secondary outcome measures of the two groups.

	FO group		Placebo group		Adjusted difference of FO vs. placebo over 12 weeks	*p^a^*
	Baseline	12 week	Baseline	12 week		
**Intention to treat (*n* = 54)**						
Handgrip (right), kg	59.3 ± 13.4	58.2 ± 14.5	63.0 ± 16.6	61.3 ± 21.0	−0.53 (−9.41, 8.35)	0.730
Handgrip (left), kg	56.0 ± 13.2	54.5 ± 12.2	59.9 ± 15.2	58.9 ± 18.8	−0.32 (−9.01, 8.37)	0.810
ALM/height*^b^*, kg/m^2^	5.4 ± 0.6	5.5 ± 0.8	5.7 ± 1.0	5.6 ± 1.1	0.19 (−0.30, 0.67)	0.479
ALM/weight × 100	23.4 ± 3.2	23.5 ± 3.7	24.3 ± 3.1	23.9 ± 3.9	1.03 (−0.88, 2.94)	0.305
Total fat percent, %	39.6 ± 6.4	39.2 ± 6.1	37.9 ± 5.3	38.7 ± 6.5	−1.15 (−3.63, 1.32)	0.424
Trunk fat percent, %	40.7 ± 6.7	40.5 ± 6.9	39.7 ± 5.0	40.6 ± 7.1	1.10 (−4.04, 1.84)	0.521
Creatinine, mg/dl	0.69 ± 0.12	0.69 ± 0.15	0.67 ± 0.20	0.70 ± 0.17	0.01 (−0.07, 0.10)	0.738
Pyruvate, μM	21.9 [17.2−27.7]	28.1 [21.0−32.1]	21.1 [18.3−32.2]	28.6 [16.2−39.8]	−	0.741
Lactate, mmol/L	1.20 ± 0.41	1.28 ± 0.55	1.25 ± 0.57	1.17 ± 0.67	−0.14 (−0.55, 0.27)	0.330
EQ-5D-3L	0.903 ± 0.119	0.945 ± 0.088	0.927 ± 0.080	0.909 ± 0.138	0.03 (−0.05, 0.11)	0.448
EQ-VAS	76.7 ± 16.3	75.5 ± 18.3	77.0 ± 13.4	73.6 ± 18.3	1.34 (−8.13, 10.81)	0.775
**Per protocol (*n* = 49)**						
Handgrip (right), kg	59.6 ± 13.6	56.0 ± 13.5	63.4 ± 16.9	64.9 ± 16.5	−5.09 (−11.14, 0.97)	0.098
Handgrip (left), kg	56.0 ± 13.4	55.1 ± 10.2	59.8 ± 15.6	61.3 ± 15.5	−3.38 (−8.03, 1.27)	0.150
ALM/height*^b^*, kg/m^2^	5.46 ± 0.62	5.54 ± 0.62*^b^*	5.67 ± 1.00	5.66 ± 1.08	0.12 (−0.25, 0.50)	0.504
ALM/weight x 100	23.6 ± 2.9	23.8 ± 2.9	24.2 ± 3.2	24.1 ± 3.7	0.81 (−0.63, 2.25)	0.263
Total fat percent,%	39.0 ± 5.9	38.9 ± 5.8	37.8 ± 5.3	37.7 ± 5.6	0.07 (−0.99, 1.13)	0.896
Trunk fat percent, %	40.2 ± 6.2	39.9 ± 6.0	39.6 ± 5.1	39.2 ± 5.4	0.29 (−1.14, 1.71)	0.686
Creatinine, mg/dl	0.70 ± 0.12	0.70 ± 0.14	0.68 ± 0.21	0.72 ± 0.16	0.01 (−0.06, 0.08)	0.874
Pyruvate, μM	21.9 (17.2−27.7)	27.5 (21.0−32.1)	25.0 (18.7−33.4)	28.6 (19.7−38.7)	−	0.899
Lactate, mmol/L	1.20 ± 0.42	1.27 ± 0.51	1.26 ± 0.57	1.23 ± 0.51	−0.23 (−0.58, 0.12)	0.195
EQ-5D-3L	0.906 ± 0.121	0.954 ± 0.070	0.934 ± 0.073	0.941 ± 0.096	0.02 (−0.05, 0.08)	0.568
EQ-VAS	77.1 ± 16.4	77.0 ± 14.5	78.0 ± 13.0	77.8 ± 13.0	−0.55 (−7.92, 6.82)	0.880

Values are mean ± SD, median [IQR], or mean (95% CI) unless otherwise indicated. FO, Fermented Oyster Extract; ALM, appendicular lean mass; EQ-5D-3L, EuroQol-5 dimensions-3-levels; VAS, visual analog scale. ^a^ANCOVA or ranked ANCOVA adjusted for age, sex, each baseline value, and the changes from baseline in dietary total caloric intake and protein intake as covariates over the 12-week period. ^b^P < 0.05 by paired t-test or Wilcoxon signed-rank test within each group.

### Safety

All subjects completed the protocol without adverse symptoms or serious adverse events. None of the participants in the two groups complained of adverse effects. Baseline concentrations of aspartate aminotransferase and alanine aminotransferase (ALT) *concentrations* in the placebo group were higher than those in the FO group (*P* = 0.040 and *P* = 0.002, respectively) in the ITT analysis, and ALT concentrations in the placebo group was higher than that in the FO group (*P* = 0.003) in the PP analysis, but within the normal range. After 12 weeks of the trial, no significant changes in BP, the concentrations of liver enzymes, fasting glucose, or creatinine were observed between the two groups. Moreover, HOMA-IR, free fatty acid, hs-CRP, CK, and malondialdehyde concentrations remained unchanged in both groups over the 12 weeks of the trial (Table 5).

**TABLE 5:** Laboratory findings evaluating adverse effects and additional benefits profile.

	FO group		Placebo group		Adjusted difference of FO vs. placebo 12 weeks	*p^a^*
	Baseline	12 week	Baseline	12 week		
**Intention to treat (*n* = 54)**						
Systolic BP, mmHg	124.4 ± 13.9	124.9 ± 14.0	126.9 ± 14.0	123.5 ± 14.2	1.53 (−7.48, 10.53)	0.619
Diastolic BP, mmHg	78.5.4 ± 10.2	79.3 ± 8.9	82.4 ± 10.3	80.8 ± 9.7	−0.23 (−5.74, 5.28)	0.754
AST, IU/L	22.9 ± 4.4	23.1 ± 7.0	25.3 ± 4.0*^b^*	24.6 ± 5.0	2.08 (−2.33, 6.48)	0.367
ALT, IU/L	16.6 ± 4.7	19.6 ± 13.6	20.7 ± 7.8*^b^*	18.6 ± 11.1	6.95 (−2.39, 16.29)	0.190
Glucose, mg/dL	94.0 [87.0−97.0]	92.0 [83.0−101.0]	92.0 [83.0−101.0]	92.0 [87.0−99.0]	−	0.246
Insulin, μU/mL	4.8 [3.8−6.8]	5.1 [4.1−6.4]	4.1 [2.4−7.0]	4.9 [2.8−7.4]	−	0.464
HOMA-IR	1.13 [0.89−1.44]	1.15 [0.95−1.71]	0.98 [0.53−1.59]	1.12 [0.67−1.71]	−	0.380
hs-CRP, mg/dl	0.05 [0.03−0.07]	0.04 [0.03−0.08]	0.04 [0.03−0.10]	0.04 [0.02−0.08]	−	0.644
FFA, mg/dL	311.7 ± 131.0	338.2 ± 173.2	357.9 ± 162.6	312.9 ± 204.9	100.25 (−41.13, 241.62)	0.165
Creatine kinase, U/L	25.1 ± 13.8	24.9 ± 17.4	27.0 ± 15.8	26.0 ± 13.2	−0.25 (−10.91, 10.33)	0.823
Malondialdehyde, μM	3.11 ± 0.78	3.19 ± 0.72	3.23 ± 0.82	3.15 ± 0.78	−0.02 (−0.53, 0.49)	0.810
**Per protocol (*n* = 49)**						
Systolic BP, mmHg	124.4 ± 14.2	124.4 ± 13.8	126.6 ± 15.1	124.0 ± 14.2	0.60 (−7.99, 9.18)	0.889
Diastolic BP, mmHg	78.2 ± 10.2	78.9 ± 8.5	82.3 ± 11.1	81.3 ± 9.1	−1.34 (−6.07, 3.38)	0.569
AST, IU/L	23.0 ± 4.3	23.2 ± 7.0	25.2 ± 4.3	25.6 ± 3.7	1.15 (−2.91, 5.21)	0.571
ALT, IU/L	16.1 ± 4.0	19.6 ± 13.6	21.6 ± 7.9*^c^*	21.1 ± 5.8	4.23 (−4.18, 12.64)	0.316
Glucose, mg/dL	94.0 [87.0−97.0]	92.5 [83.0−101.0)	92.0 (83.0−101.0)	93.0 (84.0−99.8)	−	0.270
Insulin, μU/mL	4.7 [3.8−6.6]	5.1 [4.1−6.4]	4.1 [2.5−7.0]	4.9 [2.8−6.6]	−	0.357
HOMA-IR	1.12 [0.89−1.37]	1.17 [0.95−1.71]	0.98 [0.53−1.88]	1.08 [0.67−1.64]	−	0.281
hs-CRP, mg/dl	0.04 [0.02−0.09]	0.04 [0.03−0.08]	0.05 [0.03−0.07]	0.03 [0.02−0.08]	−	0.532
FFA, mg/dL	311.7 ± 131.9	335.9 ± 173.0	362.3 ± 161.0	351.4 ± 170.2	61.44 (−63.84, 186.73)	0.328
Creatine kinase, U/L	25.8 ± 13.5	25.3 ± 16.5	28.0 ± 16.7	26.8 ± 11.5	−0.99 (−10.73, 8.75)	0.838
Malondialdehyde, μM	3.08 ± 0.78	3.19 ± 0.70	3.24 ± 0.88	3.14 ± 0.76	−0.04 (−0.51, 0.43)	0.810

Values are mean ± SD, median [IQR] or mean (95% CI) unless otherwise indicated. FO, Fermented Oyster Extract; BP, blood pressure; AST, aspartate aminotransferase; ALT, alanine aminotransferase; HOMA-IR, Homeostatic Model Assessment for Insulin Resistance; hs-CRP, high-sensitivity C-reactive protein; FFA, free fatty acids. ^a^ANCOVA or ranked ANCOVA adjusted for age, sex, each baseline value, and the changes from baseline in dietary total caloric intake and protein intake as covariates over the 12-week period. ^b^P < 0.05. ^c^P < 0.005 by paired t-test or Wilcoxon signed-rank test within each group.

## Discussion

Oysters are one of the world’s major seafood products, eaten either raw or cooked by heating or fermenting with salt. Oysters are nutritionally excellent as they are relatively rich in protein and contain minerals such as calcium and zinc, taurine, and various essential amino acids (6). Marine foods, including oysters, contain GABA. GABA is a non-protein amino acid that acts as a major inhibitory neurotransmitter in the mammalian central nervous system and contributes to motor control, growth hormone secretion, and even emotional regulation. To promote their use as functional food, marine foods are fermented using lactic acid bacteria to enhance the GABA content (21, 22). Bioactive substances for lactic acid bacteria fermentation include probiotics, prebiotics, synbiotics, and recently postbiotics (22, 23).

In a preclinical study, the muscle protein expression levels of succinate dehydrogenase, phospho-peroxisome proliferator-activated receptor gamma coactivator-1α, and adenosine monophosphate-activated protein kinase related to mitochondrial metabolism were found to be elevated in mouse myoblast C2C12 cells (ATCC, CRL-1772 cell line) following the administration of 10 and 50 μg/mL of FO. It is known that citrate plays an important role in the energy metabolism of mitochondria in muscle tissue, and its content increases when oxidative stress increases during exercise. While the citrate concentration increased in the control group, the citrate content in the muscle tissue was significantly decreased in rotarod-trained mice following the administration of 100 and 200 μg/mL of FO for 4 weeks (9). In a previous pilot study, when ten postmenopausal women were given 1,000 mg of FO per day for 8 weeks, GH and knee flexion strength were significantly increased compared to the corresponding values in 11 postmenopausal women taking a placebo. In addition, body fat significantly decreased in the treatment group compared to that in the placebo group, but muscle mass and IGF-1 concentrations did not change (11). There was no statement on whether informed consent was given by study subjects, and Institutional Review Board approval was obtained in this study. In a previous pilot study using another GABA-rich marine food, ten postmenopausal women were administered 1,000 mg of GABA-enriched sea tangle fermented with *Lactobacillus brevis* BJ20 per day for 8 weeks; GH was found to increased compared to that in the control group. However, body fat, muscle strength, and IGF-1 did not change (24). Both studies had limitations in that the study period was relatively short (8 weeks), the number of study subjects was small, and they included only on women. In addition, dietary calories and physical activity levels were not monitored during the study period.

On the contrary, the current study was conducted for 12 weeks. The number of subjects for whom statistical power was calculated based on a prior study (19) was included. Both men and women were included, and the supplement dosage was slightly increased. Additionally, dietary calories and physical activity levels were evaluated at the study’s beginning and end of the study. However, in this study, there was no difference between the control and FO groups in the change in muscle strength or GH after 12 weeks unlike the results of a previous pilot study (11). The reason for no difference between the two groups in the changes in muscle strength or GH during 12 weeks in this study could be partially explained by the different study periods and the different distribution of study subjects from the previous study. The results were the same when subgroup analysis was performed only for women.

The present study has some limitations, including the lack of biological confirmation to determine the mechanism of action of FO supplementation. Furthermore, this study focused only on middle-aged and elderly subjects with relatively low muscle mass, and thus the effect of FO on people with different conditions or of varying age groups is still unknown. In addition, this study was conducted at a single center. Lastly, the nutritional intake in this study was assessed by 24-h dietary recall; this information may not therefore represent the usual everyday diet of participants.

Despite these limitations, the current study is still considered valuable for the following reasons. Firstly, to the best of our knowledge, this is the first well-designed clinical study to examine the efficacy and tolerability of FO supplementation in adults with relatively low muscle mass. Another advantage of this study is that it evaluated participants’ physical activity through a walking log, and found that combining FO supplementation with regular walking did not remarkably improve muscle function compared to regular walking alone.

## Conclusion

In conclusion, unlike a pilot study, this study concluded that FO supplementation did not improve muscle function in adults with relatively low muscle mass. There was also no increase in concentrations of GH or IGF-1. Twelve-week FO supplementation did not cause any toxicity or severe adverse effect. However, more replication studies are needed to validate the research findings on this topic and determine the effect of FO supplementation in adults with relatively low muscle mass.

## Data availability statement

The original contributions presented in the study are included in the article, further inquiries can be directed to the corresponding authors.

## Ethics statement

The studies involving human participants were reviewed and approved by the Ethics Committee of the Institutional Review Board at Pusan National University Yangsan Hospital (IRB No. 02-2019-013, July 1, 2019). The patients/participants provided their written informed consent to participate in this study.

## Author contributions

SL contributed to the conceptualization of the study, carried out the formal analysis of the data, and coordinated and supervised the entire project. YL and SL designed the methodology of the work, had an active role in the process of participant recruitment and data acquisition, contributed to the validation of results, worked together for data curation, wrote the work’s draft, and reviewed the final document. Both authors contributed to the article and approved the submitted version.

## Footnotes

1
http://www.r-project.org/
